# Tolerance of citrus plants to the combination of high temperatures and drought is associated to the increase in transpiration modulated by a reduction in abscisic acid levels

**DOI:** 10.1186/s12870-016-0791-7

**Published:** 2016-04-27

**Authors:** Sara I. Zandalinas, Rosa M. Rivero, Vicente Martínez, Aurelio Gómez-Cadenas, Vicent Arbona

**Affiliations:** Department Ciències Agràries i del Medi Natural, Universitat Jaume I, E-12071 Castelló de la Plana, Spain; Departament de Nutrición Vegetal, Centro de Edafología Aplicada del Segura, Consejo Superior de Investigaciones Científicas, 30100 Murcia, Spain

**Keywords:** Carrizo citrange, Cleopatra mandarin, Combined stress conditions, Heat, Hormone regulation, Salicylic acid

## Abstract

**Background:**

In natural environments, several adverse environmental conditions occur simultaneously constituting a unique stress factor. In this work, physiological parameters and the hormonal regulation of Carrizo citrange and Cleopatra mandarin, two citrus genotypes, in response to the combined action of high temperatures and water deprivation were studied. The objective was to characterize particular responses to the stress combination.

**Results:**

Experiments indicated that Carrizo citrange is more tolerant to the stress combination than Cleopatra mandarin. Furthermore, an experimental design spanning 24 h stress duration, heat stress applied alone induced higher stomatal conductance and transpiration in both genotypes whereas combined water deprivation partially counteracted this response. Comparing both genotypes, Carrizo citrange showed higher phostosystem-II efficiency and lower oxidative damage than Cleopatra mandarin. Hormonal profiling in leaves revealed that salicylic acid (SA) accumulated in response to individual stresses but to a higher extent in samples subjected to the combination of heat and drought (showing an additive response). SA accumulation correlated with the up-regulation of pathogenesis-related gene 2 (CsPR2), as a downstream response. On the contrary, abscisic acid (ABA) accumulation was higher in water-stressed plants followed by that observed in plants under stress combination. ABA signaling in these plants was confirmed by the expression of responsive to ABA-related gene 18 (CsRAB18). Modulation of ABA levels was likely carried out by the induction of 9-neoxanthin cis-epoxicarotenoid dioxygenase (CsNCED) and ABA 8’-hydroxylase (CsCYP707A) while conversion to ABA-glycosyl ester (ABAGE) was a less prominent process despite the strong induction of ABA O-glycosyl transferase (CsAOG).

**Conclusions:**

Cleopatra mandarin is more susceptible to the combination of high temperatures and water deprivation than Carrizo citrange. This is likely a result of a higher transpiration rate in Carrizo that could allow a more efficient cooling of leaf surface ensuring optimal CO_2_ intake. Hence, SA induction in Cleopatra was not sufficient to protect PSII from photoinhibition, resulting in higher malondialdehyde (MDA) build-up. Inhibition of ABA accumulation during heat stress and combined stresses was achieved primarily through the up-regulation of CsCYP707A leading to phaseic acid (PA) and dehydrophaseic acid (DPA) production. To sum up, data indicate that specific physiological responses to the combination of heat and drought exist in citrus. In addition, these responses are differently modulated depending on the particular stress tolerance of citrus genotypes.

**Electronic supplementary material:**

The online version of this article (doi:10.1186/s12870-016-0791-7) contains supplementary material, which is available to authorized users.

## Background

Plants respond to adverse environmental challenges by activating specific molecular and physiological changes to minimize damage. The great majority of studies focusing on plant stress tolerance have considered a single stress condition. However, under field conditions, several abiotic stress situations are most likely to occur simultaneously constituting a unique new stress condition and not a mere additive combination of the effects of the individual stress factors [1, 2]. Therefore, the future development of broad-spectrum stress-tolerant plants will require the understanding of the responses to multiple abiotic threats and, hence, new experimental approaches have to be developed in order to mimic stress combinations [2]. Particularly, drought and elevated temperatures represent the most frequent abiotic stress combination occurring in natural environments [1]. This situation has important detrimental effects on plant growth and productivity [3–5]. Additionally, plant responses to a combination of drought and high temperatures have been suggested to be exclusive and different from plant responses to drought or heat stress applied individually [6–8].

Plant responses to external stimuli are mainly mediated by phytohormones, whose involvement in abiotic stress has been deeply studied [9–12]. Under drought or high salinity, abscisic acid (ABA) seems to be an important stress-signaling hormone [13, 14], involved in the regulation of stomatal closure, synthesis of compatible osmolytes and up-regulation of genes leading to adaptive responses. Increase of ABA levels is accompanied by the up-regulation of 9-neoxanthin cis-epoxicarotenoid dioxygenase (NCED) that converts 9-neoxanthin to xanthoxin and is considered the bottleneck in ABA biosynthesis. Inactivation of ABA is achieved by its cleavage to 8’-OH-ABA catalyzed by an ABA 8’-hydroxylase (CYP707A) and this compound is converted spontaneously to phaseic acid (PA) and subsequently to dehydrophaseic acid (DPA) as main degradation products. Additionally, another pathway for removing active ABA pools is the conjugation to hexoses by an ABA O-glycosyl transferase (AOG) yielding ABA-glycosyl ester (ABAGE) [15]. Finally, active ABA can be released after cleavage of ABAGE by an ABAGE β-glycosidase (BG18) [16] and Additional file 1A.

Salicylic acid (SA) has been associated to defense responses against biotrophic pathogens [17]. However, recent studies have suggested that SA also plays an important role in abiotic stress-induced signaling and tolerance [11, 18]. Particularly, it has been proposed that SA may induce thermotolerance in several plant species [19–22]. Studies in *Arabidopsis* mutants suggest that SA-signaling pathways involved in the response to biotic stresses overlap with those promoting basal thermotolerance. In this sense, pathogenesis-related (PR) genes are not only induced by biotic stresses but also in response to high temperatures [21]. This plant hormone is synthesized from chorismate in a reaction catalyzed by isochorismate synthase (ICS) and subsequently by isochorismate pyruvate lyase. In addition, SA is also synthesized from phenylalanine and the key enzyme catalyzing this reaction is phenylalanine ammonia lyase (PAL) [23] and Additional file 1B. SA accumulation induced by stress, exogenous application or genetic manipulation has been associated to positive responses against high temperature stress in different plant species such as poplar [24], *Agrostis stolonifera* [25], *Avena sativa* [26] and grapevine [27]. The benefits of SA accumulation seem to be associated to an improvement in antioxidant activity and the protection of the photosynthetic machinery avoiding electron leakage [28]. In addition, an improvement in the responses to other abiotic stress conditions such as salinity, drought or chilling have been reported [11].

Despite these advances in hormonal physiology, it is still unclear how different signaling pathways with such clear roles interact to induce defense responses in plants when several stress conditions concur. For instance, stomatal responses, which are essential in acclimation to abiotic stress conditions, have been recently associated to the interaction of reactive oxygen species (ROS), ABA and Ca^2+^ waves [29]. Briefly, upon ABA sensing, mediated by pyrabactin resistance1/PYR-like/regulatory components of ABA receptors (PYR/PYL/RCAR) and protein phosphatases 2C (PP2Cs), sucrose non-fermenting 1-related protein kinases (SnRKs) 2.3 is released and phosphorylates slow anion channel-associated 1 (SLAC1), a membrane ion channel that mediates anion release from guard cells promoting stomatal closure. In addition, SnRKs2.3 also phosphorylates and activates a plasma-bound NADPH oxidase (RBOH) involved in O_2_^•-^ production that is dismutated into H_2_O_2_ by apoplastic superoxide dismutases. The elevated ROS levels enhance ABA signaling through inhibition of PP2Cs and activate influx Ca^2+^ channels, increasing its cytosolic concentration. Subsequently, this Ca^2+^ accumulation contributes to inhibit ion influx into guard cells and maintain stomatal closure. This mechanism is in line with apoplastic ROS modulating the responsiveness of guard cells to ABA [30]. Moreover, ROS have been shown to promote ABA biosynthesis and inhibit its degradation, resulting in an increase of endogenous ABA levels [29].

In this work, we aimed to study the physiological and hormonal responses to drought, heat and their combination in two citrus genotypes with contrasting stress tolerance, Carrizo citrange and Cleopatra mandarin, and link tolerance responses to a differential SA and ABA accumulation and signaling.

## Methods

### Plant material and growth conditions

True-to-type Carrizo citrange (*Poncirus trifoliata* L. Raf. x *Citrus sinensis* L. Osb.) and Cleopatra mandarin (*Citrus reshni* Hort. Ex Tan.) plants were purchased from an authorized commercial nursery (Beniplant S.L., Penyíscola, Spain). One-year-old seedlings of both citrus genotypes were placed in 0.6-L plastic pots filled with perlite and watered three times a week with 0.5 L of a half-strength Hoagland solution in greenhouse conditions (natural photoperiod and day and night temperature averaging 25.0 ± 3.0 °C and 18.0 ± 3.0 °C, respectively). Later, plants of both genotypes were maintained for 2 weeks in growth chambers to acclimate to a 16-h photoperiod at 300 μmol m^−2^ s^−1^ at 25 °C and relative moisture at approximately 80 %. Temperature and relative moisture were recorded regularly with a portable USB datalogger (OM-EL-WIN-USB, Omega, New Jersey, USA).

### Stress treatments and experimental designs

To evaluate heat stress tolerance, Carrizo citrange and Cleopatra mandarin seedlings were subjected to 40 °C for 10 days and the number of intact sprouts (sprouts with no visual symptoms of damage: wilting, bronzing and/or abscission at gentle touch) was recorded regularly. Similarly, citrus plants were maintained at 40 °C while imposing water withdrawal to investigate the effects of the stress combination. Percentage of intact sprouts was calculated at 0, 2, 4, 6, 8 and 10 days after imposing stress treatments.

Additionally, we designed a 24-h experiment in which severe drought, imposed by transplanting plants to dry perlite, was applied alone or in combination with high temperatures (40 °C). Prior to imposition of drought regime, heat stress (HS) was applied for 7 days to a group of well-watered Carrizo and Cleopatra plants whereas another group was maintained at 25 °C. Thereby, we established four experimental groups of each genotype: well-watered plants at 25 °C (CT) and 40 °C (HS) and plants subjected to drought at 25 °C (WS) and at 40 °C (WS + HS). Leaf tissue was sampled at 24 h after subjecting plants to both stresses.

### Physiological parameters

Gas exchange and chlorophyll fluorescence parameters were measured in parallel on plants of each treatment between 9:00 and 11:00 h. Leaf gas exchange parameters were measured with a LCpro + portable infrared gas analyzer (ADC bioscientific Ltd., Hoddesdon, UK) under ambient CO_2_ and moisture. Supplemental light was provided by a PAR lamp at 1000 μmol m^−2^ s^−1^ photon flux density and air flow was set at 150 μmol mol^−1^. After instrument stabilization, ten measurements were taken on three mature leaves (from an intermediate position on the stem) in three replicate plants from each genotype and treatment. Quantum yield (Φ_PSII_) and maximum efficiency of photosystem II (PSII) photochemistry, as F_v_/F_m_ ratio, were analyzed on the same leaves and plants using a portable fluorometer (FluorPen FP-MAX 100, Photon Systems Instruments, Czech Republic).

### Proline analysis

0.05 g ground, frozen leaf tissue was extracted in 5 ml of 3 % sulfosalicylic acid (Panreac, Barcelona, Spain) by sonication for 30 min. After centrifugation at 4000 g for 20 min at 4 °C, extracts were assayed for proline as described by Bates and others [31] with slight modifications. Briefly, 1 ml of the supernatant was mixed with 1 ml of glacial acetic acid and ninhydrin reagent (Panreac) in a 1:1 (v:v) ratio. The reaction mixture was incubated in a water bath at 100 °C for 1 h. After centrifuging at 2000 g for 5 min at 4 °C, absorbance was read at 520 nm. A standard curve was performed with standard proline (Sigma-Aldrich, St. Louis, MO, USA).

### Leaf water status

Leaf relative water content (RWC) was measured using adjacent leaves, which were immediately weighed to obtain a leaf fresh mass (M_f_). Then, leaves were placed in a beaker with water and kept overnight in the dark, allowing leaves to become fully hydrated. Leaves were reweighed to obtain turgid mass (M_t_) and dried at 80 °C for 48 h to obtain dry mass (M_d_). Finally, RWC was calculated as [(M_f_ - M_d_) × (M_t_ - M_d_)^−1^] × 100 according to [32].

### Malondialdehyde analysis

Malondialdehyde (MDA) content was measured following the procedure of [33] with some modifications. Ground frozen leaf tissue (0.2 g approximately) were homogenized in 2 mL 80 % cold ethanol by sonication for 30 min. Homogenates were centrifuged 12000 g for 10 min and different aliquots of the supernatant were mixed either with 20 % trichloroacetic acid or with a mixture of 20 % trichloroacetic acid and 0.5 % thiobarbituric acid. Both mixtures were incubated in a water bath at 90 °C for 1 h. After that, samples were cooled in an ice bath and centrifuged at 2000 g for 5 min at 4 °C. The absorbance at 440, 534 and 600 nm of the supernatant was read against a blank.

### Plant hormonal analysis

Hormone extraction and analysis were carried out as described in [34] with few modifications. Shortly, for ABA, PA, DPA and SA extractions, 0.3 g of ground frozen leaf tissue was extracted in 2 mL of ultrapure water after spiking with 50 ng of [^2^H_6_]-ABA, [^13^C_6_]-SA and [^2^H_3_]-PA in a ball mill (MillMix20, Domel, Železniki, Slovenija). After centrifugation at 4000 g at 4 °C for 10 mins, supernatants were recovered and pH adjusted to 3 with 30 % acetic acid. For ABAGE extraction, the aqueous layer was recovered and after adding 0.1 M sodium hydroxide, was incubated in a water bath at 60 °C for 30 min. Then, samples were cooled in an ice bath and 50 ng of [^2^H_6_]-ABA was added. pH was adjusted to 3 with 0.5 % chlorhydric acid. All water extracts were partitioned twice against 2 mL of diethyl-ether and then the organic layer was recovered and evaporated under vacuum in a centrifuge concentrator (Speed Vac, Jouan, Saint Herblain Cedex, France). Once dried, the residue was resuspended in a 10:90 methanol:water solution by gentle sonication. The resulting solution was filtered through 0.22 μm polytetrafluoroethylene membrane syringe filters (Albet S.A., Barcelona, Spain) and directly injected into an ultra performance liquid chromatography system (Acquity SDS, Waters Corp., Milford, MA, USA). Chromatographic separations were carried out on a reversed-phase C18 column (Gravity, 50 × 2.1 mm 1.8-μm particle size, Macherey-Nagel GmbH, Germany) using a methanol:water (both supplemented with 0.1 % acetic acid) gradient at a flow rate of 300 μL min^−1^. Hormones were quantified with a triple quadrupole mass spectrometer (Micromass, Manchester, UK) connected online to the output of the column though an orthogonal Z-spray electrospray ion source.

### Total RNA isolation and cDNA synthesis

About 100 mg of ground Carrizo and Cleopatra leaf tissue was used to isolate total RNA by RNeasy Mini Kit (Qiagen) following the manufacturer’s instructions. Then, 5 μg RNA was treated with RNase-free DNase (Promega Biotech Ibérica, SL. Madrid, Spain) according to the manufacturer in order to remove genomic DNA contamination. The integrity of the RNA was assessed by agarose gel electrophoresis and ethidium bromide staining. Total RNA concentration was determined using spectrophotometric analysis (NanoDrop, Thermo Scientific, Wilmington, DE, USA), and the purity was assessed from the ratio of absorbance readings at 260 and 280 nm. Reverse transcription was carried out from 1 μg of total RNA using Primescript RT reagent with oligo(dT) primer (Takara Bio, Inc. Japan).

### qRT-PCR analyses

Gene-specific primers were designed with primer3plus (http://www.bioinformatics.nl/cgi-bin/primer3plus/primer3plus.cgi) using orthologous sequences retrieved from *Citrus sinensis* genome (http:\\www.phytozome.org) (Additional file 2: Table S1). Designed primers were then evaluated with IDT-oligoanalyzer tools (http://eu.idtdna.com/analyzer/applications/oligoanalyzer/) following parameters: Tm around 60 °C, amplicon length of 125 to 200 bp, primer length of 18 to 22 nucleotides with an optimum at 20 nucleotides and, finally, a GC content of 45 to 55 %. Amplicon specificity was evaluated by agarose gel electrophoresis and by melting-curve analyses. The expression of all genes was normalized against the expression of two endogenous control genes (tubulin and actin). Relative expression levels were calculated by using REST software [35], comparing the expression of the gene at a particular time point to a common reference sample from the tissue at the first time point and then expression values were expressed as fold change of control values for each stress conditions. qRT-PCR analyses were performed in a StepOne Real-Time PCR system (Applied Biosystems, CA, USA). The reaction mixture contained 1 μL of cDNA, 5 μL of SYBR Green (Applied Biosystems) and 1 μM of each gene-specific primer pair in a final volume of 10 μL. The following thermal profile was set for all amplifications: 95 °C for 30 s followed by 40 cycles of 95 °C for 5 s and 60 °C for 30 s. Three technical replicates were analyzed on each biological replicate.

### Statistical analyses

Statistics were evaluated with the Statgraphics Plus v.5.1. software (Statistical Graphics Corp., Herndon, VA, United States). Data are means of three independent determinations and were subjected to one- or two-way analysis of variance (ANOVA) followed by Tukey posthoc test (*p* < 0.05) when a significant difference was detected. 

## Results

### Tolerance of Carrizo and Cleopatra plants to high temperatures and combined heat and drought

**Fig. 1:**
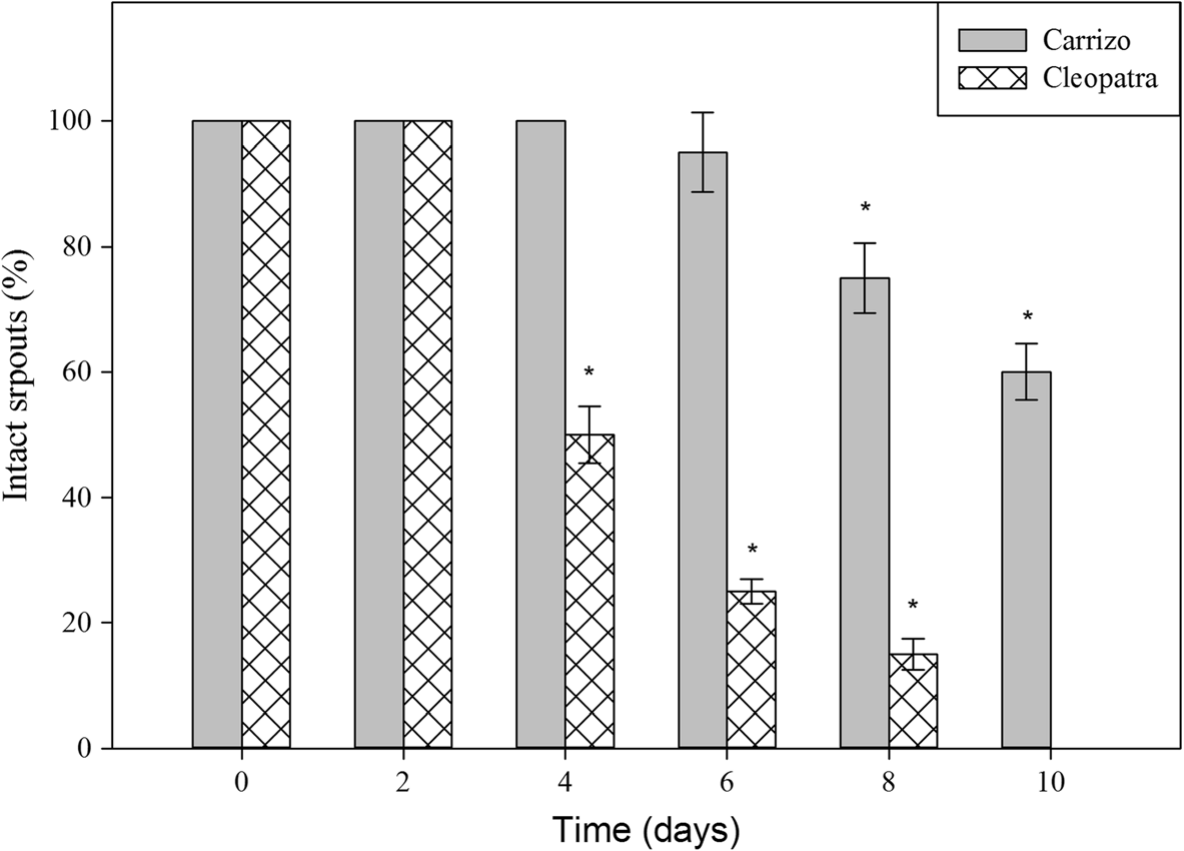
Phenotypic traits of citrus plants in response to a combination of drought and heat stress. Intact sprouts (%) of Carrizo and Cleopatra seedlings subjected to drought and heat stress (40 °C) in combination for 10 days. For each genotype, asterisks denote statistical significance with respect to initial values at *p* ≤ 0.05

The citrus genotypes used in this study, Carrizo citrange and Cleopatra mandarin, were chosen due to their differences in tolerance to different abiotic stress conditions [36]. However, little is known about their ability to tolerate high temperatures. Hence, the relative tolerance to high temperature of the two genotypes employed in this study was firstly investigated. To accomplish this, both genotypes were subjected to continuous heat stress (40 °C) for 10 days. The ability to produce new flushes and maintain sprouts healthy throughout the experimental period was taken as a tolerance trait. All seedlings growing at 40 °C showed an intense flushing of new sprouts compared to those grown at normal temperature (25 °C) (Additional file 3A and B, D and E). However, as the experiment progressed, new sprouts in Cleopatra started browning and withering (Additional file 3E-F), affecting more than 70 % of the new flushes after 6 days of treatment (Additional file 3G). On the contrary, new sprouts appearing on Carrizo did not show any damage symptom throughout the experimental period (Additional file 3B-C). Only at the end of the experimental process, 20 % of the new flushes in Carrizo showed symptoms of damage (Additional file 3G). These results clearly evidenced the higher tolerance of Carrizo to high temperatures compared to Cleopatra. Moreover, we also recorded the number of intact sprouts in Carrizo and Cleopatra seedlings subjected to a combination of heat (40 °C) and water deprivation for 10 days (Fig. 1). After 4 days of treatment, only 50 % of new sprouts in Cleopatra plants remained unaffected whereas all sprouts on Carrizo looked healthy. At 8 days of treatment, Carrizo sprouts started showing symptoms of damage, but a 75 % still remained intact. At this point, however, only 15 % of Cleopatra sprouts showed no apparent damage. At the end of the experiment (10 days), 60 % of Carrizo sprouts still remained unaffected by stress treatment, while all Cleopatra sprouts were severely damaged, thus evidencing a higher ability of Carrizo to tolerate drought and heat applied in combination. To this respect, tolerance to high temperatures of both genotypes greatly mirrored tolerance to heat and water stress combination.

### Effects on osmotic status under drought, heat and combined stresses

**Fig. 2:**
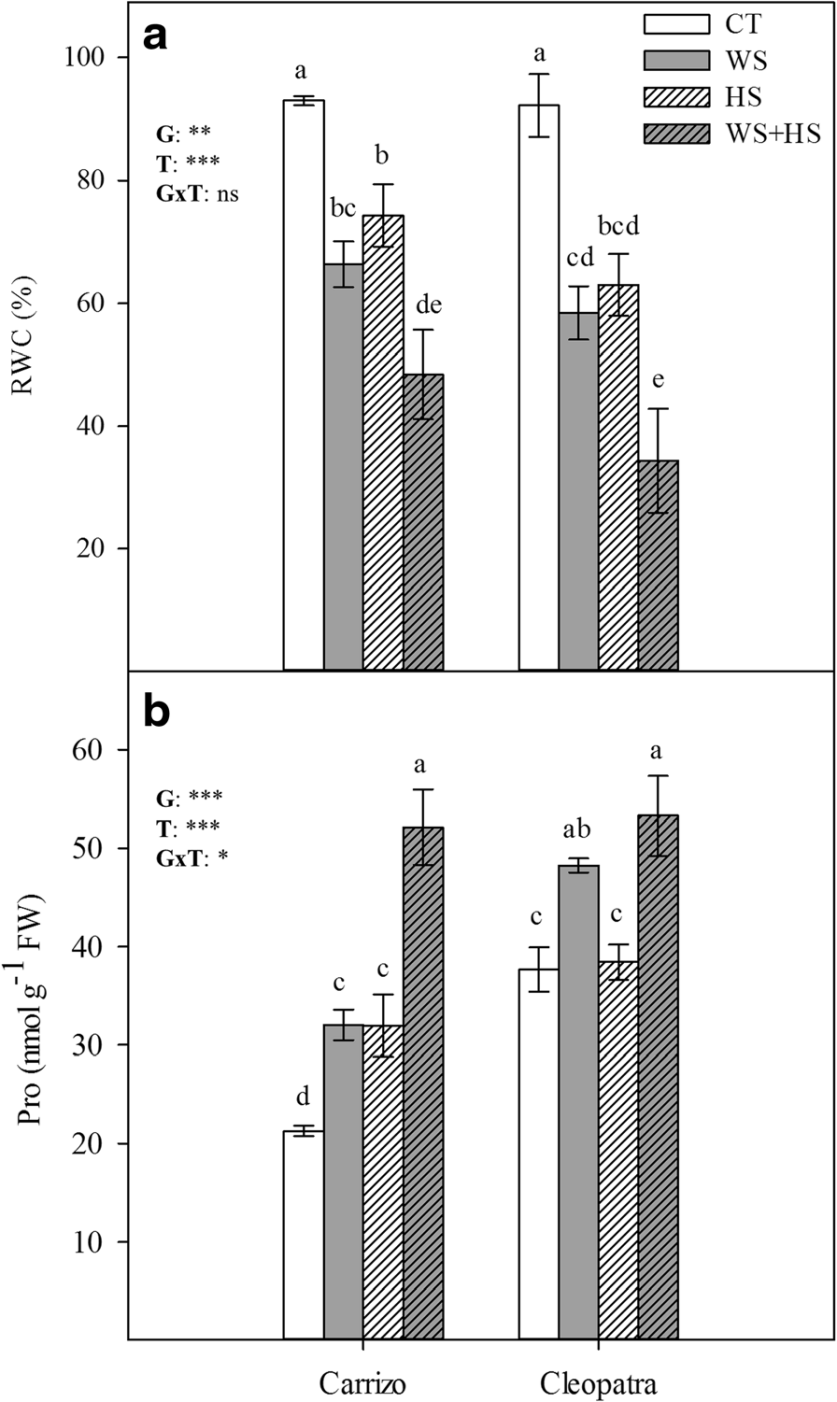
Relative water content (RWC) (**a**) and proline concentration (**b**) in Carrizo and Cleopatra plants subjected to drought (WS), heat (HS) and their combination (WS + HS). Different letters denote statistical significance at *p* ≤ 0.05. G: genotypes; T: stress treatment; GxT: interaction genotype x stress treatment. *P < 0.05; **P < 0.01; ***P < 0.001; ns: no statistical differences

Leaf RWC was measured for each genotype and stress treatment (Fig. 2a). In the conditions assayed in this work, abiotic stress conditions induced similar significant decreases in RWC in both genotypes. When applied individually, water stress and heat stress induced similar decreases in leaf RWC in plants of Carrizo and Cleopatra (60–70 % of control values). Interestingly, stress combination had an additive effect on this parameter. Therefore, WS + HS plants exhibited the most dramatic reduction in leaf RWC showing levels that were 48.4 % and 34.3 % of control values in Carrizo and Cleopatra, respectively.

In line with the observed variations in RWC, endogenous proline levels, as a compatible osmolyte, were inspected (Fig. 2b). In response to WS, proline levels increased by 1.4-fold and 1.3-fold, respect to control values in Carrizo and Cleopatra, respectively. Moreover, HS induced an accumulation of proline in leaves of Carrizo whereas in Cleopatra, it had no significant effect. As for RWC, the stress combination had an additive effect on proline levels, inducing the highest leaf proline accumulation of all treatments, an average of 52.7 nmol g^−1^ fresh weigh (FW) in both genotypes (Fig. 2b). Interestingly, proline levels in leaves of non-stressed Cleopatra seedlings were higher than in Carrizo (37.5 nmol g^−1^ FW versus 21.0 nmol g^−1^ FW, respectively). A correlation analysis between RWC and proline was performed, showing *R* values of 0.8065 and 0.6504 in Carrizo and Cleopatra, respectively, and *p*-values of <0.01 in both citrus genotypes.

### Leaf gas exchange and fluorescence parameters under drought, heat and combined stresses

**Fig. 3:**
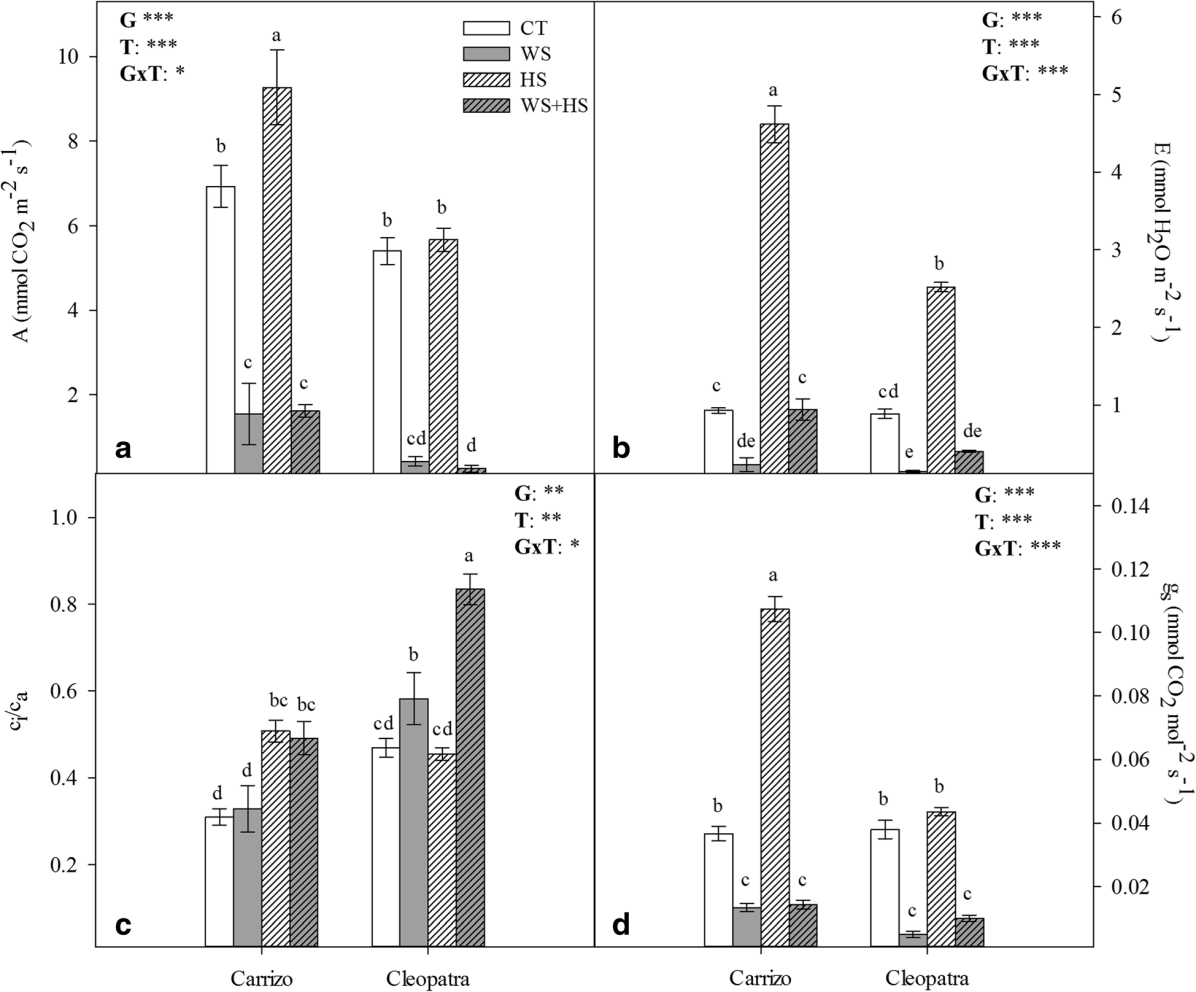
Gas exchange parameters in citrus plants subjected to different stress treatments. Leaf photosynthetic rate, A (**a**), transpiration, E (**b**), ratio of substomatal-to-ambient CO_2_, C_i_/C_a_ (**c**), stomatal conductance, g_s_ (**d**) in Carrizo and Cleopatra plants subjected to drought (WS), heat (HS) and their combination (WS + HS). Different letters denote statistical significance at *p* ≤ 0.05. G: genotypes; T: stress treatment; GxT: interaction genotype x stress treatment. *P < 0.05; **P < 0.01; ***P < 0.001; ns: no statistical differences

**Fig. 4:**
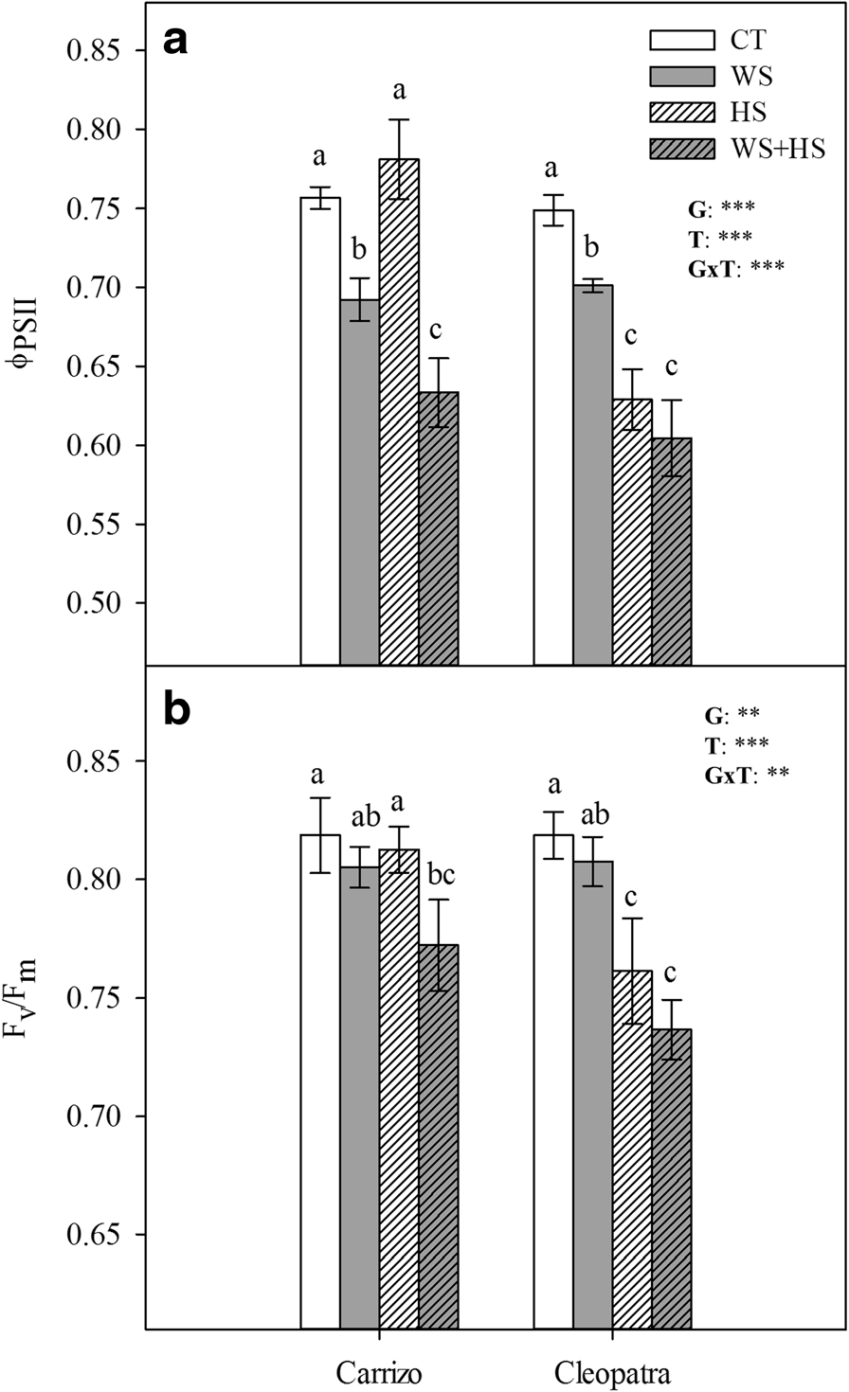
Chlorophyll fluorescence parameters in citrus plants subjected to different stress treatments. Quantum efficiency (Φ_PSII_) (**a**) and maximum efficiency of PSII photochemistry (F_v_/F_m_ ratio) (**b**) in Carrizo and Cleopatra plants subjected to drought (WS), heat (HS) and their combination (WS + HS). Different letters denote statistical significance at *p* ≤ 0.05. G: genotypes; T: stress treatment; GxT: interaction genotype x stress treatment. *P < 0.05; **P < 0.01; ***P < 0.001; ns: no statistical differences

Leaf photosynthetic rate (A), transpiration (E), carboxylative efficiency (in terms of substomatal-to-ambient CO_2_, (C_i_/C_a_) ratio) and stomatal conductance (g_s_) were measured in both genotypes (Fig. 3). In general, WS and WS + HS reduced A, E and g_s_ parameters compared to unstressed plants mainly in Cleopatra. On the other hand, HS increased these parameters, especially in Carrizo, almost doubling Cleopatra levels in some cases (Fig. 3a, b and d). However, this effect of HS was counteracted by WS under WS + HS conditions. Plants subjected to stress combination showed similar gas exchange values to those obtained for WS plants in both genotypes. Additionally, carboxylative efficiency was affected by HS and stress combination in Carrizo. In Cleopatra, C_i_/C_a_ ratio increased slightly in response to WS. However, stress combination had a pronounced effect on carboxylative efficiency in this genotype (Fig. 3c). In addition to this, we measured the quantum efficiency of PSII photochemistry (Φ_PSII_) and the maximum efficiency of PSII photochemistry (F_v_/F_m_ ratio) that correlated with gas exchange parameters (Fig. 4). In Carrizo, WS had a predominant effect over HS on electron transport between photosystems (Φ_PSII_) whereas WS, HS or their combination was detrimental for this parameter in Cleopatra, having HS a more pronounced effect than WS alone. Moreover, F_v_/F_m_ measurements mostly mirrored results obtained for Φ_PSII_ showing a negative effect of HS applied alone only in Cleopatra whereas stress combination affected both genotypes similarly.

### MDA accumulation

Lipid peroxidation was measured in terms of MDA content. According to data (Table 1), MDA accumulated significantly in Carrizo leaves only in response to WS + HS. In Cleopatra leaves, MDA content increased in the three experimental conditions but higher levels were found under the combined effect of WS + HS, reaching values of 234.2 nmol g^−1^ FW, representing three times the MDA content of control plants (85.1 nmol g^−1^ FW).

**Table 1:** Malondialdehyde (MDA) concentration in citrus plants subjected to stress treaments. drought (WS), heat (HS) and their combination (WS + HS)

MDA content (nmol g^−1^ FW)		
Stress condition	Carrizo	Cleopatra
CT	112.83^cd^ ± 3.46	85.11^d^ ± 7.15
WS	120.89^c^ ± 1.30	118.16^cd^ ± 5.95
HS	106.82^cd^ ±1.15	116.15^cd^ ±3.26
WS + HS	160.02^b^ ± 0.48	234.21^a^ ±16.55
G	*	
T	***	
GxT	***	

Different letters denote statistical significance at *p* ≤ 0.05. G: genotypes; T: stress treatment; GxT: interaction genotype x stress treatment. *P < 0.05; **P < 0.01; ***P < 0.001; ns: no statistical differences

### SA metabolism and signaling under drought, heat and combined stresses

**Fig. 5:**
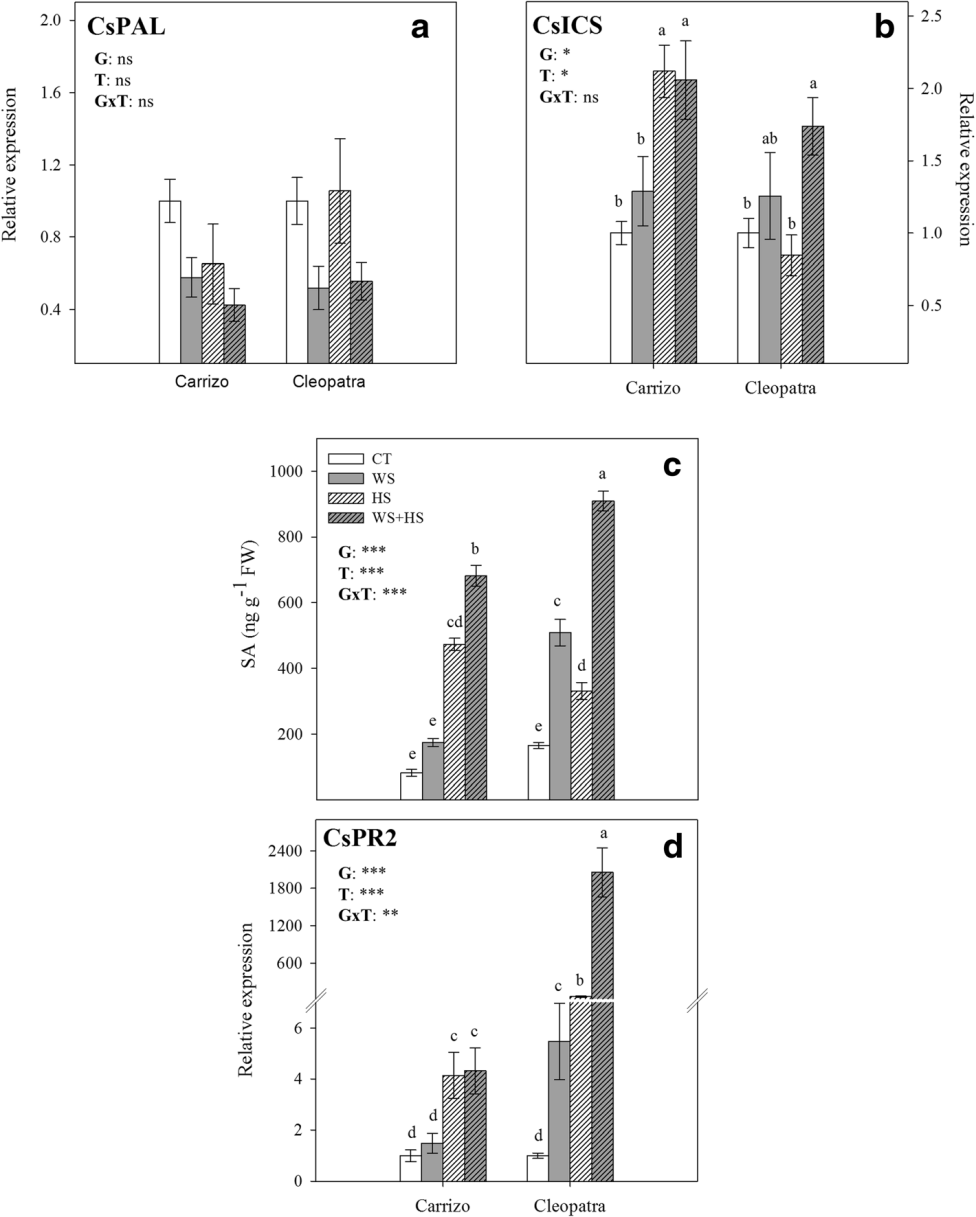
Effect of the different stress treatments on metabolism and signaling of SA. CsPAL (**a**) and CsICS (**b**) relative expression, SA concentration (**c**) and CsPR2 (**d**) relative expression in Carrizo and Cleopatra plants subjected to drought (WS), heat (HS) and their combination (WS + HS). Different letters denote statistical significance at *p* ≤ 0.05. G: genotypes; T: stress treatment; GxT: interaction genotype x stress treatment. *P < 0.05; **P < 0.01; ***P < 0.001; ns: no statistical differences

We measured SA levels in citrus leaves subjected to drought, heat stress and the combination of both stresses (Fig. 5c). WS and HS and the combination of stresses increased SA levels in leaves of both genotypes respect to CT values, but higher levels were always observed in WS + HS plants. Interestingly, Cleopatra plants under WS + HS and WS showed SA levels 2.2-fold and 3.0-fold respectively higher than Carrizo. Moreover, we analyzed the relative expression of CsPAL and CsICS, two genes involved in SA biosynthetic pathways [23] in response to WS, HS and WS + HS. No statistical differences were found between genotypes or stress treatments in CsPAL transcript levels (Fig. 5a) whereas CsICS expression was significantly altered during HS and WS + HS in Carrizo leaves and during WS + HS in Cleopatra (Fig. 5b), showing the highest expression levels, respectively.

To confirm SA signaling, we also analyzed the expression of CsPR2, a protein functioning as β-1,3-glucanase activity involved in defense against biotrophic pathogens that is induced by SA [37]. CsPR2 transcript abundance correlated with SA accumulation in leaves of citrus, being strongly induced in leaves of WS + HS Cleopatra plants, showing the greatest SA levels. In general, abiotic stress induced higher SA build-up in Cleopatra than in Carrizo and hence a stronger CsPR2 expression. Moreover, in Carrizo, only treatments involving heat (HS and WS + HS) resulted in a significant increase in CsPR2 transcript levels whereas all abiotic stress treatments induced expression of this gene in Cleopatra plants (Fig. 5d).

### ABA metabolism under drought, heat and combined stresses

**Fig. 6:**
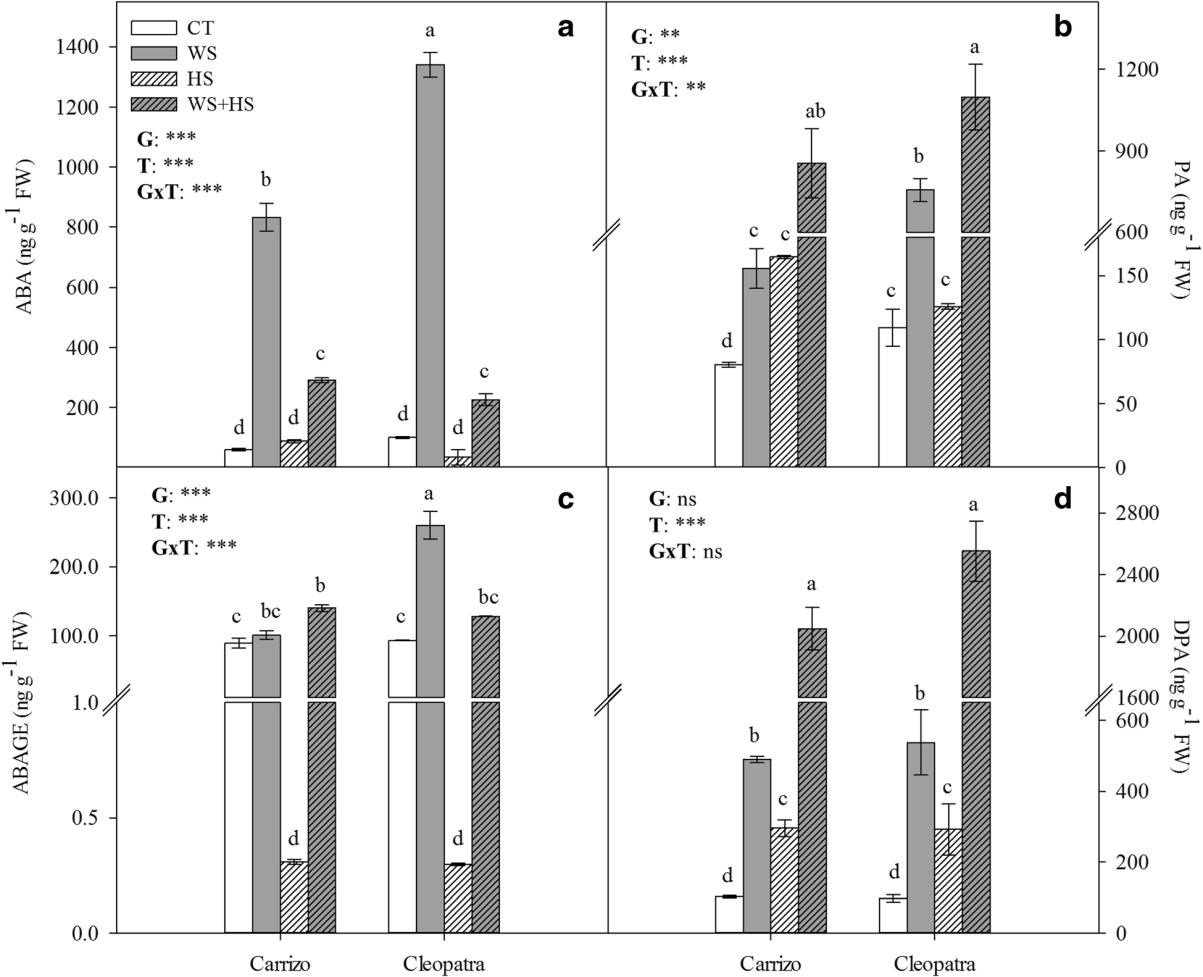
ABA, ABAGE, PA and DPA levels in citrus plants subjected to different stress treatments. ABA (**a**), ABAGE (**c**), PA (**b**) and DPA (**d**) levels in Carrizo and Cleopatra plants subjected to drought (WS), heat (HS) and their combination (WS + HS). Different letters denote statistical significance at *p* ≤ 0.05. G: genotypes; T: stress treatment; GxT: interaction genotype x stress treatment. *P < 0.05; **P < 0.01; ***P < 0.001; ns: no statistical differences

Analysis of ABA showed that WS and, to a much lower extent, WS + HS combination increased ABA levels in both citrus genotypes, reaching about 831.9 and 1340.1, and 290.9 and 225.7 ng g^−1^ FW, respectively (Fig. 6a). Conversely, HS did not have any significant influence on ABA concentration in any of the two genotypes.

To further investigate ABA metabolism in these stress conditions, concentration of PA and DPA as main ABA degradation products (Fig. 6b-d) as well as the accumulation of ABAGE (Fig. 6c) were measured. WS increased PA and DPA levels in leaves of both citrus genotypes but only Cleopatra exhibited a significant increment of ABAGE. In addition, HS induced the accumulation of DPA and reduced ABAGE content below control levels in both genotypes. During heat stress treatment, only Carrizo showed a significant PA accumulation (Fig. 6b). Finally, WS + HS combination resulted in a strong accumulation of PA and DPA in both citrus genotypes that was significantly higher than in WS treatment. Stress combination slightly induced ABAGE accumulation only in Carrizo.

### Genes involved in ABA metabolism and signal transduction

**Fig. 7:**
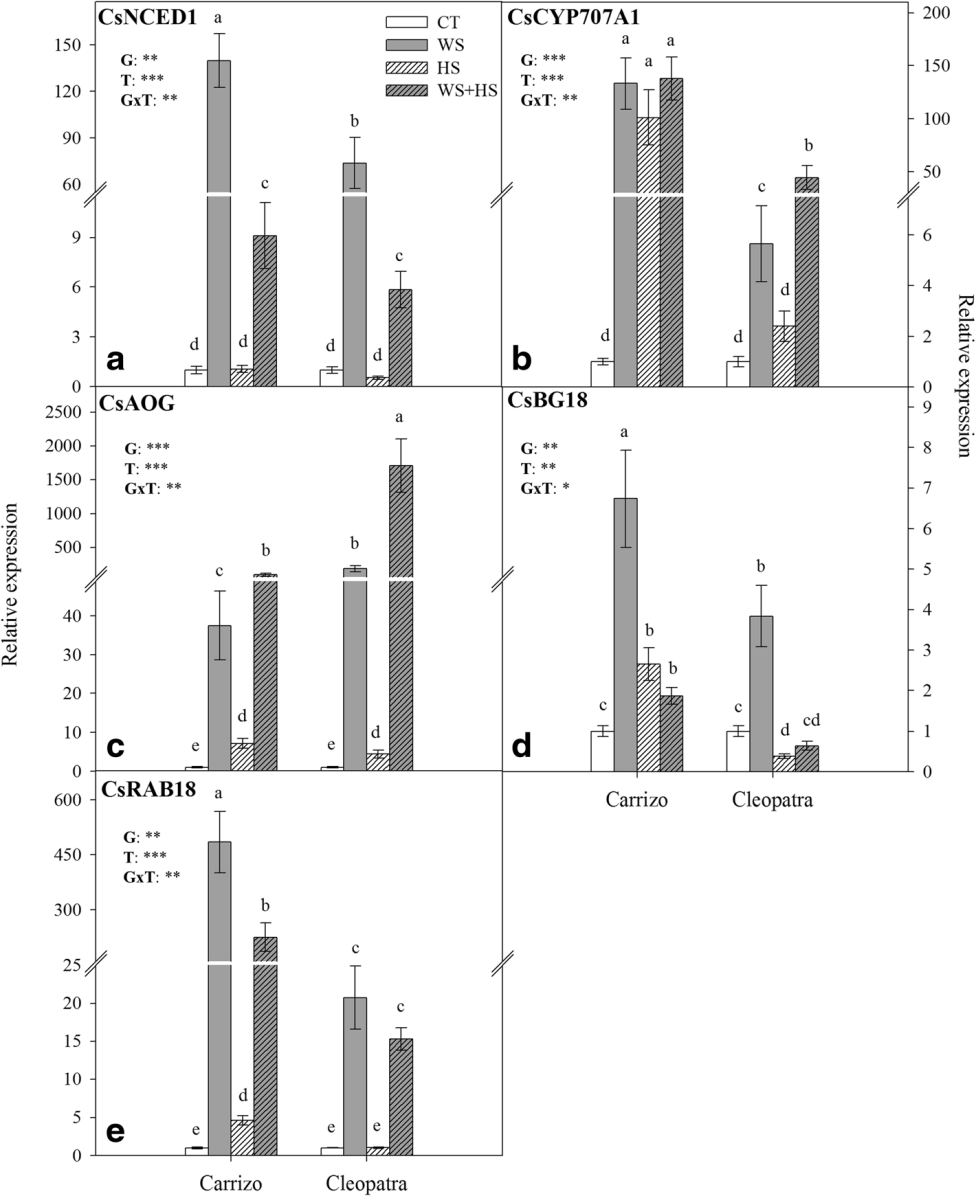
Expression of genes involved in ABA biosynthesis, catabolism, conjugation and signaling in citrus plants subjected to different stress treatments. Relative expression of ABA-biosynthetic gene CsNCED1 (**a**), ABA-related catabolism gene CsCYP707A1 (**b**), ABA-related conjugation genes CsAOG and CsBG18 (**c**-**d**) and ABA-signaling gene CsRAB18 (**e**) in leaves of Carrizo and Cleopatra plants in response to drought (WS), heat (HS) and their combination (WS + HS). Different letters denote statistical significance at *p* ≤ 0.05. G: genotypes; T: stress treatment; GxT: interaction genotype x stress treatment. *P < 0.05; **P < 0.01; ***P < 0.001; ns: no statistical differences

To understand how ABA metabolism is modulated under the stress conditions assayed, the relative expression of genes encoding proteins involved in ABA biosynthesis, catabolism and conjugation were analyzed. In addition, responsive to ABA-related gene 18 (CsRAB18) expression was measured to confirm the occurrence of ABA signal transduction (Fig. 7).

When WS was applied alone or in combination, the expression of CsNCED1 was induced in leaves of Carrizo and, to a lower extent, in Cleopatra. But, on the contrary, HS did not change the expression of this gene in any of the genotypes studied (Fig. 7a). Hence, in stress combination, HS always counteracted the WS-dependent induction of CsNCED1. Additionally, CsCYP707A1 expression was up-regulated in all stress treatments but showed different induction profiles depending on the genotype. Overall, expression was higher in Carrizo than in Cleopatra but, conversely to CsNCED1, stress treatments involving heat (HS and WS + HS) also induced CsCYP707A1 expression (Fig. 7b). No differences were recorded for CsCYP707A1 expression values among stress treatments in Carrizo. In Cleopatra, WS increased CsCYP707A1 expression up to 6-fold while HS had a more moderate impact and WS + HS combination induced its expression up to 50-fold. In the ABA conjugation pathway, CsAOG expression pattern was similar to that of CsNCED1 but showing a more intense up-regulation in Cleopatra than in Carrizo upon WS + HS imposition and a significant slight induction by HS alone (Fig. 7c). Although CsAOG gene was primarily induced by WS, stress combination had an additive effect on its expression showing values of 90.2 and 1704.9 in Carrizo and Cleopatra, respectively (Fig. 7c). Moreover, CsBG18 expression was up-regulated primarily in response to WS in both genotypes and in response to HS and WS + HS only in Carrizo (Fig. 7d); in Cleopatra HS induced a significant down-regulation whereas stress combination had no significant effect.

Additionally, stress signal transduction mediated by ABA was assessed by studying the expression of CsRAB18, encoding a dehydrin protein, as an ABA-responsive gene. The expression pattern of this gene followed greatly that shown by CsNCED1 (Fig. 7a) and also that exhibited by ABA levels (Fig. 6a). Accumulation of CsRAB18 transcripts in leaves of both genotypes was observed mainly in response to WS and WS + HS and it was more pronounced in Carrizo. In this genotype, HS induced a slight increment in CsRAB18 expression, while no changes were observed in Cleopatra (Fig. 7e).

## Discussion

In the field, plants are often subjected to a combination of different abiotic stress conditions. Most research projects have focused on plant responses to a single stress factor under controlled environment. However, it is predicted that responses of plants to a combination of stress conditions could not be inferred simply from the study of each individual stress [1, 7]. For this reason, there is a need to understand the nature of responses to multiple stresses in order to develop plants more tolerant to environmental cues in a climate change scenario. In this context, drought and heat represent two stress conditions that are expected to increase their incidence in the next 50–100 years, drastically affecting global agricultural systems (IPCC, 2007). In the Mediterranean climate, summer drought is accompanied by high temperatures that limit crop plant growth, development and production. In the present research, we studied the relative tolerance and the physiological and molecular responses to heat, drought and a combination of both stress conditions of two citrus genotypes: Carrizo citrange and Cleopatra mandarin. These two citrus species show contrasting ability to tolerate different abiotic stress conditions. It has been reported that Cleopatra is more tolerant to drought and salinity than Carrizo, whereas the latter is more tolerant to soil flooding conditions [36]. However, information on citrus responses to heat stress is scarce. For this reason, in a preliminary study, we assessed heat susceptibility of both citrus genotypes by analyzing sprout emission and survival of plants subjected to a 10-day period of high temperatures (40 °C) alone and combined with water withdrawal (Additional file 3 and Fig. 1). Heat stress had a detrimental effect on Cleopatra sprout survival whereas Carrizo sprouts remained visibly healthy until the end of the experiment (Additional file 3), indicating that Carrizo is more tolerant to heat stress than Cleopatra. Similarly, Carrizo showed higher ability to tolerate heat stress combined with drought since 60 % of sprouts remained intact after 10 days of stress combination. On the other hand, all sprouts were damaged in Cleopatra by the end of the experiment, showing only 50 % of intact sprouts after 4 days of WS + HS (Fig. 1). Nevertheless, it is worthwhile noting that at day 8 all sprouts in Cleopatra plants were damaged in response to HS (Additional file 3), whereas in response to WS + HS, 12.5 % of sprouts still remained healthy on the same date (Fig. 1). This apparent inconsistency could be explained by the effect of water stress and high temperature combination on stomatal closure. Cleopatra plants have been previously reported to be tolerant to salt stress due to a fast decrease in transpiration rate during the osmotic phase of salinity that prevents build-up of chloride ions [38]. In this sense, the similar effect caused by WS would lead to a sharp decrease in transpiration rate during WS + HS conditions respect to HS that would prevent further desiccation. Hence, WS would act buffering the damaging effects of HS subsequently yielding a significantly higher percentage of intact sprouts in WS + HS plants.

### Physiological responses of citrus to WS, HS and their combination

Water deprivation induced similar decreases of RWC in plants of both genotypes, indicating that the impact of WS was identical to both genotypes. On the other hand, HS incremented plant transpiration in both citrus genotypes. The combination of both stress conditions resulted in drastic decreases in leaf RWC, probably due to the additive effects of the individual stresses (drought induced water loss and high temperatures increased transpiration). In this sense, the accumulation of the compatible osmolyte proline was also highest in WS + HS treatments. Proline is an osmotically active molecule [39–43] although it is also accumulated in response to other types of stresses. Therefore, besides its known role as a compatible osmolyte, proline exhibits many other protective effects, including maintenance of redox balance and radical scavenging, maintenance of protein native structure acting as a molecular chaperonin enhancing the activities of different enzymes and contributing to lessen cell membrane damage [40, 44]. Under our conditions, proline accumulation was associated to water loss induced by soil drought, the elevated transpiration rates associated to the high temperatures or both. To show this association, we have performed a correlation analysis between RWC and proline, obtaining *p*-values <0.01 and *R* values of 0.8065, 0.6504 for Carrizo and Cleopatra, respectively. As previously shown ([36] and references therein), different basal levels of proline between genotypes, as well as other protective and regulatory mechanisms, could be behind the higher tolerance of Cleopatra plants to drought. In well-watered plants of this genotype, proline levels are higher than in those of Carrizo. This seems to protect cells against stress for an extended period without significant additional increases in proline concentration [36]. Hence, a stronger stress pressure or longer treatment periods are required to cause further proline accumulation. Therefore, the high constitutive levels of proline prevent subsequent osmolyte biosynthesis until more severe stress conditions are reached, hence altering the linear relationship between the phenotypic trait (RWC) and the biochemical response.

To further characterize the responses to stress combination, gas exchange and chlorophyll fluorescence parameters were analyzed. As expected, WS alone induced stomatal closure, reducing transpiration and net photosynthetic rate in both citrus genotypes. On the contrary, HS induced an increase of transpiration probably oriented to decrease leaf surface temperature via evaporative cooling. However, HS had a different impact on transpiration in Carrizo and Cleopatra. In this latter genotype, high temperature treatment resulted in a lower increase of transpiration that did not have a concomitant impact on A or g_s_. These different gas exchange responses could constitute a physiological advantage of Carrizo over Cleopatra since, as previously reported [8], higher transpiration rate could be linked to a lower leaf temperature. Moreover, stress also had an effect on net CO_2_ assimilation, which was more pronounced in Cleopatra than in Carrizo, as evidenced by C_i_/C_a_ ratio. In this sense, the capability of plants to modulate leaf gas exchange parameters and maintain optimal CO_2_ assimilation rates under heat stress is directly associated to high temperature stress tolerance [45]. Moreover, under stress combination (WS + HS), the effect of WS on gas exchange parameters predominated over HS indicating that induction of stomatal closure to minimize water loss prevailed over responses that could lead to a reduction of leaf surface temperature (Fig. 3). Chlorophyll fluorescence data further evidenced the detrimental influence of HS on the ability of Cleopatra plants to photosynthesize. In this genotype, PSII performance (F_v_/F_m_) and photosynthetic electron flow (Φ_PSII_) significantly decreased in both HS treatments (HS and WS + HS) while PSII values in Carrizo plants were affected only by WS + HS combination (Fig. 4). PSII, and especially the oxygen-evolving complex, is the most heat-sensitive component of the photosynthetic system [27, 45, 46]. These results are coherent with gas exchange data and further support the higher tolerance of Carrizo citrange to increased temperatures. WS + HS combination had the most detrimental effect on both genotypes evidenced by a reduction in F_v_/F_m_. In addition, the concomitant Φ_PSII_ reduction could be attributed to the lower PSII efficiency and the increased stomatal closure induced by water stress. As indicated by the parallel response of gas exchange and chlorophyll fluorescence parameters, the reduction in the ability to fix CO_2_ could be associated to impairment in the performance of PSII, linked to the decrease in photosynthetic electron flow and, to a lesser extent, to the drought-induced stomatal closure. In other plant systems, similar results have been reported. In *Arabidopsis thaliana*, the combination of heat and drought resulted in the simultaneous enhancement of respiration and suppression of photosynthesis. Heat stress induced stomatal opening and enhanced photorespiration whereas drought caused a suppression of photosynthesis linked to stomatal closure [7]. Similarly, tobacco plants subjected to drought exhibited a severe reduction in net photosynthetic rate while application of heat shock resulted in stomatal opening and an increase in transpiration and photorespiration but without alteration of net photosynthetic rate. Overall, the combination of drought and heat suppressed transpiration and photosynthesis but induced an increase in photorespiration rate [8]. Our data are in agreement with these reports, since combination of drought and heat affected plants in a different manner, suggesting that plants under combined stresses that could not cool their leaves by increasing transpiration as in heat stress conditions, faced a more damaging situation.

The effect on photochemistry is often linked to electron leakage and induction of oxidative damage [47]. The degree of lipid peroxidation (determined by monitoring changes in the levels of MDA) can be related to the balance between ROS production and antioxidant activity within a given cell or tissue. WS + HS combination increased MDA levels in leaves of both citrus genotypes although a higher accumulation was observed in Cleopatra respect to Carrizo seedlings (Table 1). The higher levels of MDA observed in Cleopatra leaves under the combination of WS and HS indicate a stronger incidence of oxidative damage associated to a higher ROS production and also to a less efficient ROS detoxification system, as previously shown [48]. Interestingly, these results also correlate to net photosynthetic rate and C_i_/C_a_ ratio (Fig. 3a-c) since during WS + HS, Cleopatra displayed a more pronounced reduction in net CO_2_ assimilation than Carrizo. It has been shown that ROS have an influence on ABA biosynthesis and signaling that alters Ca^2+^ influx to stomata guard cells and modulates stomatal opening [29, 30]. Hence, this higher ROS production in combination with an active ABA signaling could be behind the stomatal closure observed under WS + HS combination [29]. On the other hand, the lower MDA accumulation observed in Carrizo is compatible with a lower ROS production possibly associated to the more efficient antioxidant system of this genotype [48] and a the less sensitive photosynthetic system to abiotic stressors that is able to modulate excess photosynthetic electron input even under adverse environmental conditions [49, 50].

### Involvement of ABA and SA to the response to drought, heat and combined stresses

To our knowledge, little is known on hormonal responses of citrus plants to heat or its combination with other stress conditions. In this work, the hormonal profile revealed significant changes in ABA and SA, hormones that have been involved in abiotic stress [12, 51] and plant thermotolerance [19–22, 52], respectively.

The role of SA in plant–pathogen interactions has been extensively investigated, being involved in systemic acquired resistance (SAR), a stronger defense response mediated by the PR proteins [17, 23, 53]. In addition to defense responses, SA plays an important role in the response to abiotic stresses [11] and especially in the tolerance to high temperatures [19–22, 52]. Moreover, previous studies indicate that SA-signaling pathways involved in SAR overlap with those promoting basal thermotolerance since mutations known to affect SA-signaling in pathogen defenses also affect heat tolerance [21]. In the present study, SA levels increased in response to stresses applied individually and more prominently in both citrus genotypes subjected to WS + HS combination showing an additive output. This major SA accumulation observed during WS + HS was accompanied by a significant up-regulation of CsICS, whereas the involvement of the PAL pathway in the active SA biosynthesis could be considered marginal. This data would reinforce the idea that stress combination has a higher impact to physiology than individual stresses applied alone. Additionally, the relative expression of CsPR2, a gene induced by SA in citrus plants [37], remarkably increased in response to heat treatments (HS and WS + HS) mainly in Cleopatra leaves. This confirmed the stronger response of Cleopatra probably intended to mitigate the detrimental effects of heat stress. In previous reports, SA has been proposed to protect PSII complex [27, 54] and could be involved in the maintenance of membrane integrity during heat stress [21]. Data presented here are in agreement with these proposals as the most affected genotype, Cleopatra, also showed the strongest SA build-up and CsPR2 transcript accumulation. This genotype exhibited a stronger accumulation of MDA, therefore, requiring a higher accumulation of protective SA. Nevertheless, the higher accumulation of SA found in Cleopatra leaves under WS + HS conditions was not sufficient to prevent heat-induced membrane damage. In general, data in this study indicate that SA, as well as CsPR2 transcripts, are predominantly accumulated in response to WS + HS and could be related to the higher damage induced by the two stress situation acting together. Therefore, stress combination would represent a more damaging situation for plants than disconnected stresses.

The role of ABA on plant responses to water stress is well-known but there is not much information on its involvement in heat stress. Different stress treatments induced similar ABA accumulation patterns in both genotypes. An interesting finding is the fact that while WS induced the typical ABA accumulation in citrus tissues, HS (alone or applied in combination with WS) greatly inhibited this response, suggesting that during different abiotic stress conditions citrus leaves undergo substantially different programs regulating ABA homeostasis. Endogenous ABA levels are regulated through the coordinated action of biosynthesis, catabolism and conjugation yielding ABAGE [15, 55–57]. In our study, WS induced a strong accumulation of ABA in plants of both genotypes, coincident with stomatal closure and accompanied by a concerted up-regulation of CsNCED1, CsCYP707A1, CsAOG and CsBG18 gene expression leading to significant amounts of PA, DPA and ABAGE. On the contrary, HS did not vary either ABA levels or CsNCED1 expression in leaves of both genotypes. However, PA and DPA accumulation along with the up-regulation of CsCYP707A1 indicated an induction of ABA catabolism under high temperature conditions. Apart from catabolism, conjugation to hexoses was also activated since a significant accumulation of CsAOG transcripts was observed. Despite this up-regulation, an increment of ABAGE content during HS could not be observed. This could be associated to the fact that HS induced the ABA catabolic pathway without a concomitant induction of CsNCED1. Therefore, although CsAOG gene expression was up-regulated, there was not enough ABA to be conjugated.

Strikingly, under combination of drought and heat, ABA levels moderately increased in parallel with a moderate CsNCED1 up-regulation, much lower than that observed under WS. In this sense, HS could prevent the huge accumulation of ABA through the partial down-regulation of CsNCED1 and up-regulation of genes encoding for ABA 8’-hydroxylase and ABA glycosyl transferase. Additionally, analytical and gene expression data indicated that ABA catabolism actively participates in the reduction of hormone levels under HS and WS + HS conditions whereas conjugation to hexoses had a marginal role in the conditions assayed, despite the strong CsAOG transcript accumulation observed. Nevertheless, the role of hexose conjugation as an additional mechanism to precisely modulate active ABA levels under particular stress conditions cannot be ruled out. Despite the strong reduction in ABA levels during WS + HS respect to WS conditions, data indicate that either the remaining amount of hormone found was enough to close stomata and reduce photosynthetic rate or, as suggested previously [29], an interaction between ROS and ABA signaling cooperatively contributed to close stomata. In this sense, it has been previously discussed that treatments with H_2_O_2_ induce a reduction in stomatal aperture when ABA signaling is repressed [58]. In this sense, the increased incidence of oxidative damage observed in WS + HS conditions is an indication of an excess H_2_O_2_ production that probably participated in the reduction of gas exchange parameters as observed under WS. Moreover, CsRAB18 expression confirmed ABA signaling, which correlated with ABA content under all stress conditions. Overall, our data indicate that while WS increases ABA contents via *de novo* biosynthesis, HS would activate pathways involved in removing active hormone pools mainly through catabolism. At the physiological level, the observed inhibition of WS-induced ABA accumulation associated to HS could be a specific response aimed to down-regulate ABA signaling and increase stomatal opening and transpiration, allowing an adequate refrigeration of leaves. In line with these data, a recent report in *Arabidopsis thaliana* revealed a decrease of leaf ABA in response to HS correlated with a down-regulation of AtNCED3 expression and an up-regulation of AtCYP707A3 [59], supporting the idea that ABA reduction in photosynthetic organs is a necessary response to induce stomatal opening and subsequently to enhance transpiration as a direct response to the heat stimulus.

Our results indicate that Cleopatra is more sensitive to heat stress than Carrizo, especially when combined with drought. This higher tolerance of Carrizo could be associated to an improved leaf cooling via enhanced transpiration along with the ability to modulate photosynthetic electron flow, resulting in a lower incidence of oxidative damage. In both citrus genotypes, stress combination represents a more damaging situation than the individual stresses, inducing a higher damage to PSII leading to the accumulation of MDA. In addition, SA accumulation and signaling paralleled stress sensitivity and correlated with a higher requirement of SA-mediated protective responses, being both greater in Cleopatra mandarin than in Carrizo citrange. ABA levels increased in response to WS but not during HS and, interestingly, WS + HS resulted in lower hormone levels than drought applied alone. Furthermore, the transcriptional regulation of ABA metabolism during drought and heat applied alone and in combination pointed to a unique mechanism of response for each stress condition as reported in previous studies [1, 60]. Although it is widely accepted that NCED has a central role in the regulation of ABA levels under stress conditions, as shown in different plant species [57, 61, 62], the up-regulation of CsCYP707A1, CsAOG and CsBG18 could act cooperatively to modulate active ABA pools during WS when NCED activity is elevated. Therefore, these results indicate that the activation of ABA degradation and conjugation could contribute to fine-tune hormone levels during WS and HS.

## Conclusions

At present, information on the combined effect of heat and drought stress in citrus is rather limited. In this work, we have demonstrated the different ability of two citrus genotypes, Carrizo citrange and Cleopatra mandarin, to tolerate drought and heat applied alone or in combination. In this sense, physiological responses in terms of gas exchange parameters and chlorophyll fluorescence, along with MDA accumulation as an estimation of oxidative damage, evidenced the susceptibility of Cleopatra mandarin to combined heat and drought conditions. The different pattern of ABA accumulation (along with specific transcriptional regulation of genes involved in ABA metabolism) in response to each individual stress situation and their combination pointed to a unique mechanism of response to each stress condition. Additionally, SA levels and associated signaling positively correlated with stress sensitivity, being more pronounced in Cleopatra mandarin. Tolerance to a combination of different stress factors mimicking field conditions should be the focus of future research programs aimed to develop genetically-engineered plants with enhanced tolerance to several environmental conditions. Additionally, study of hormone crosstalk already observed in citrus and other species [63] could be also relevant under combined abiotic stress conditions leading to plant tolerance. The understanding of the underlying mechanisms of response and the existing interactions among abiotic stressors will provide valuable information for crop improvement.

### Consent to publish

Not applicable.

### Availability of data and materials

Raw data could be obtained by request to the corresponding author. The datasets supporting the conclusions of this article are included within the article and its additional files. Gene sequences are available in the Phytozome database (see Additional file 2: Table S1 and https://phytozome.jgi.doe.gov/pz/portal.html).

## Additional files

Additional file 1: Schematic diagram showing the biosynthetic and signaling pathways of ABA (A) and SA (B). Names in red are the genes analyzed in this work and the different metabolites studied are presented in black squares. (PDF 190 kb) Additional 2: Table S1. Designed primers for gene expression analyses by quantitative RT-PCR. (PDF 69 kb) Additional file 3: Effects of heat stress treatment on citrus sprouts. Carrizo control sprouts (25 °C) (A), Carrizo plants subjected to heat stress for 10 days (B), sprouts on control (right) and heat-stressed (left) Carrizo plants (C), Cleopatra control sprouts (D), Cleopatra plants subjected to heat stress for 10 days (E), sprouts on control (right) and heat-stressed (left) Cleopatra plants (F), integral sprouts (%) of Carrizo and Cleopatra seedlings subjected to heat stress for 10 days. For each genotype, asterisks denote statistical significance at *p* ≤ 0.05 respect to initial values (G). (PDF 318 kb)

## Abbreviations

A: net photosynthetic rate
ABA: abscisic acid
ABAGE: ABA-glycosyl ester
AOG: ABA O-glycosyl transferase
C_i_/C_a_: ratio of substomatal to ambient CO_2_ concentration
CYP707A: ABA 8’-hydroxylase
DPA: dehydrophaseic acid
E: Transpiration
F_v_/F_m_: maximum efficiency of photosystem II
g_s_: stomatal conductance
ICS: isochorismate synthase
MDA: malondialdehyde
NCED: 9-neoxanthin cis-epoxicarotenoid dioxygenase
PA: phaseic acid
PAL: phenylalanine ammonia lyase
PR2: pathogenesis-related gene 2
PSII: photosystem II
PP2C: protein phosphatase 2C
PYR/PYL/RCAR: pyrabactin resistance1/PYR-like/regulatory components of ABA receptor
Φ_PSII_: quantum efficiency of PSII photochemistry
RAB18: responsive to ABA-related gene 18
ROS: reactive oxygen species
RWC: relative water content
SA: salicylic acid
SnRK: sucrose non-fermenting 1-related protein kinase
