# Supplementary material for: Tolerance of citrus plants to the combination of high temperatures and drought is associated to the increase in transpiration modulated by a reduction in abscisic acid levels
Source: BMC Plant Biol. 2016 Apr 27;16:105. doi: 10.1186/s12870-016-0791-7 (PMC4848825; doi:10.1186/s12870-016-0791-7)
Supplement: Additional file 1: — Schematic diagram showing the biosynthetic and signaling pathways of ABA (A) and SA (B). Names in red are the genes analyzed in this work and the different metabolites studied are presented in black squares. (PDF 190 kb) [file 12870_2016_791_MOESM1_ESM.pdf]

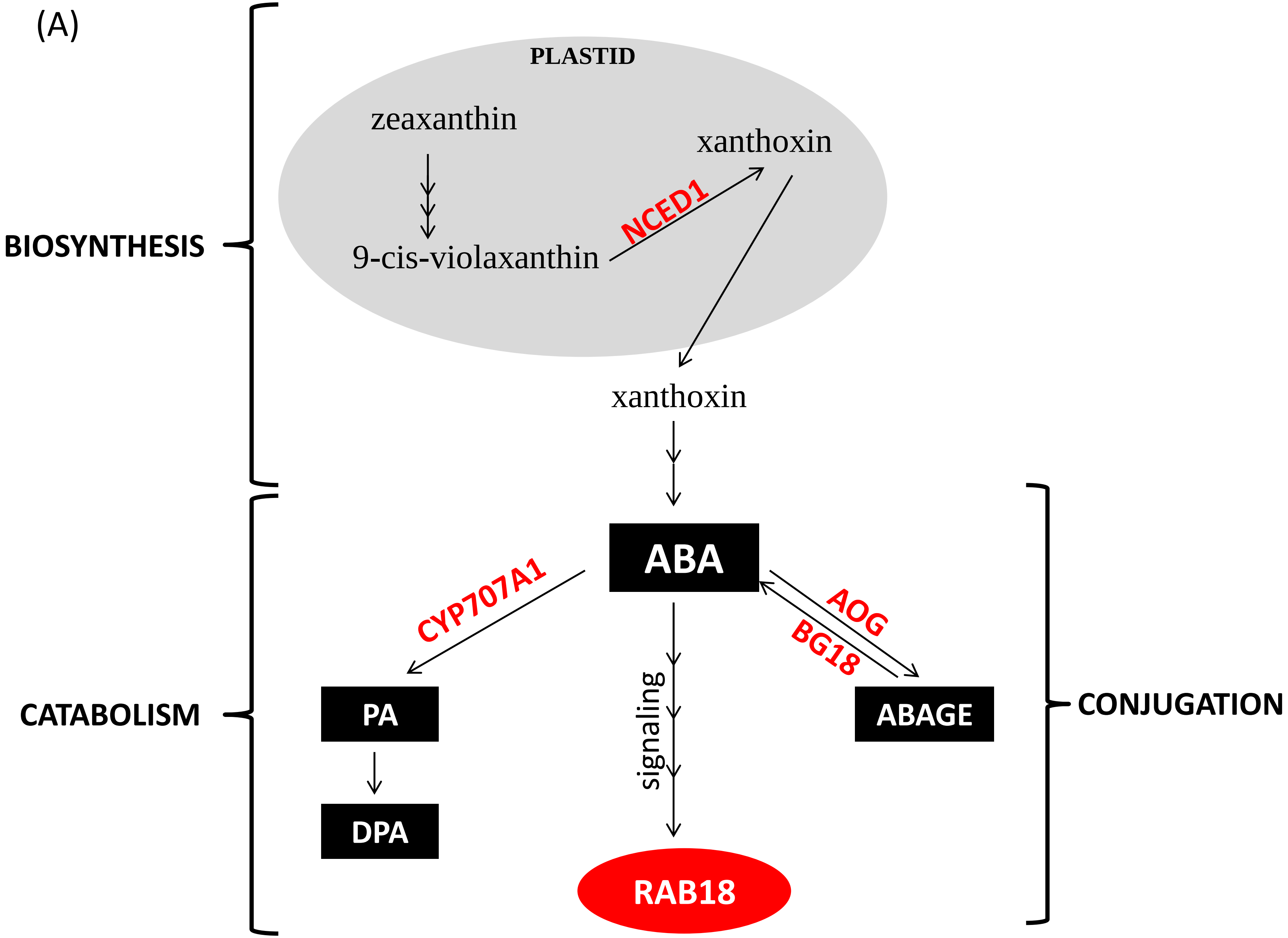
(A)
BIOSYNTHESIS
PLASTID
zeaxanthin
9-cis-violaxanthin
NCED1
xanthoxin
xanthoxin
CATABOLISM
ABA
CYP707A1
AOG
BG18
PA
DPA
signaling
ABAGE
CONJUGATION
RAB18

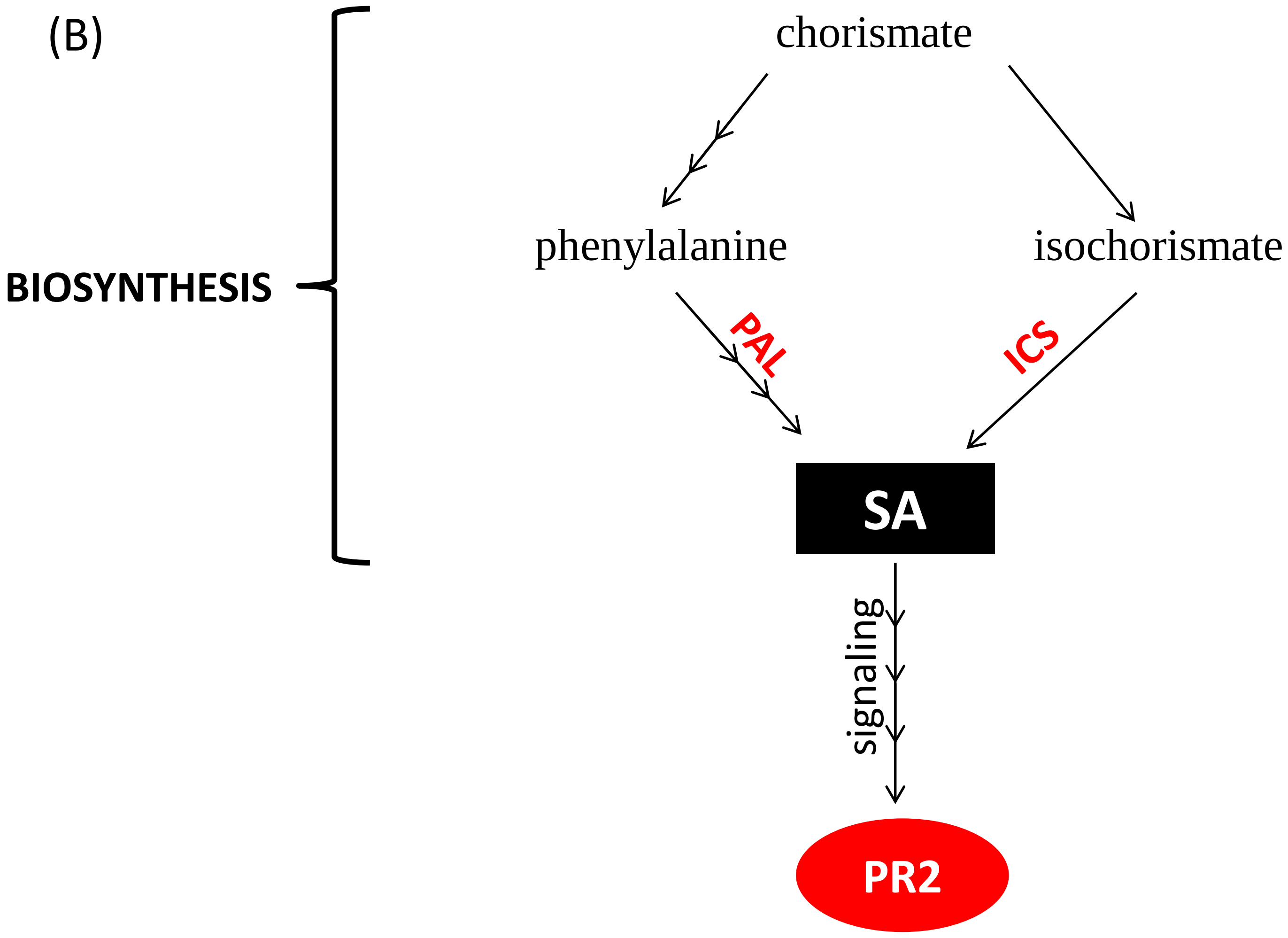

Additional file 1. Schematic diagram showing the biosynthetic and signaling pathways of ABA (A) and SA (B). Names in red are the genes analyzed in this work and different metabolites studied are presented in black squares.
