# Supplementary material for: Tolerance of citrus plants to the combination of high temperatures and drought is associated to the increase in transpiration modulated by a reduction in abscisic acid levels
Source: BMC Plant Biol. 2016 Apr 27;16:105. doi: 10.1186/s12870-016-0791-7 (PMC4848825; doi:10.1186/s12870-016-0791-7)
Supplement: Additional 2: Table S1. — Designed primers for gene expression analyses by quantitative RT-PCR. (PDF 69 kb) [file 12870_2016_791_MOESM2_ESM.pdf]

Additional File S1.- Table. Designed primers for gene expression analyses by quantitative RT-PCR.

Citrus gene	Locus	Forward / Reverse	Sequence (5'→3')	Amplicon size (bp)
**CsNCED1**	orange1.1g007379m	F	AATGCTTGGGAAGAGCCTGAG	147
		R	AGTGGACTCGCCGGTCTTTAG	
**CsCYP707A1**	orange1.1g038621m	F	TCAATGTTGCACTGCTCTCC	246
		R	CTTTGGCACCCATGAAAGAT	
**CsAOG**	orange1.1g022744m	F	CGGGTTCACTGTTTGGTCTT	176
		R	GCCTCGAGAGAAATGGATGT	
**CsBG18**	orange1.1g010588m	F	CAAGGCAAAACAGGGTGGAT	212
		R	CAGCCTCAGAGCTGGTGAAT	
**CsRAB18**	orange1.1g028210m	F	CTGAAGCTGAACGGGAGATT	195
		R	TTGTGGTGGTAGAGGTGGTG	
**CsACT**	orange1.1g037845m	F	CCCTTCCTCATGCCATTCTTC	105
		R	CGGCTGTGGTGGTAAACATG	
**CsTUB**	orange1.1g013335m	F	GGGGCAAAATGAGCACTAAA	187
		R	CGCCTGAACATCTCCTGAAT	
