# Supplementary material for: Tolerance of citrus plants to the combination of high temperatures and drought is associated to the increase in transpiration modulated by a reduction in abscisic acid levels
Source: BMC Plant Biol. 2016 Apr 27;16:105. doi: 10.1186/s12870-016-0791-7 (PMC4848825; doi:10.1186/s12870-016-0791-7)
Supplement: Additional file 3: — Effects of heat stress treatment on citrus sprouts. Carrizo control sprouts (25 °C) (A), Carrizo plants subjected to heat stress for 10 days (B), sprouts on control (right) and heat-stressed (left) Carrizo plants (C), Cleopatra control sprouts (D), Cleopatra plants subjected to heat stress for 10 days (E), sprouts on control (right) and heat-stressed (left) Cleopatra plants (F), integral sprouts (%) of Carrizo and Cleopatra seedlings subjected to heat stress for 10 days. For each genotype, asterisks denote statistical significance at p ≤ 0.05 respect to initial values (G). (PDF 318 kb) [file 12870_2016_791_MOESM3_ESM.pdf]

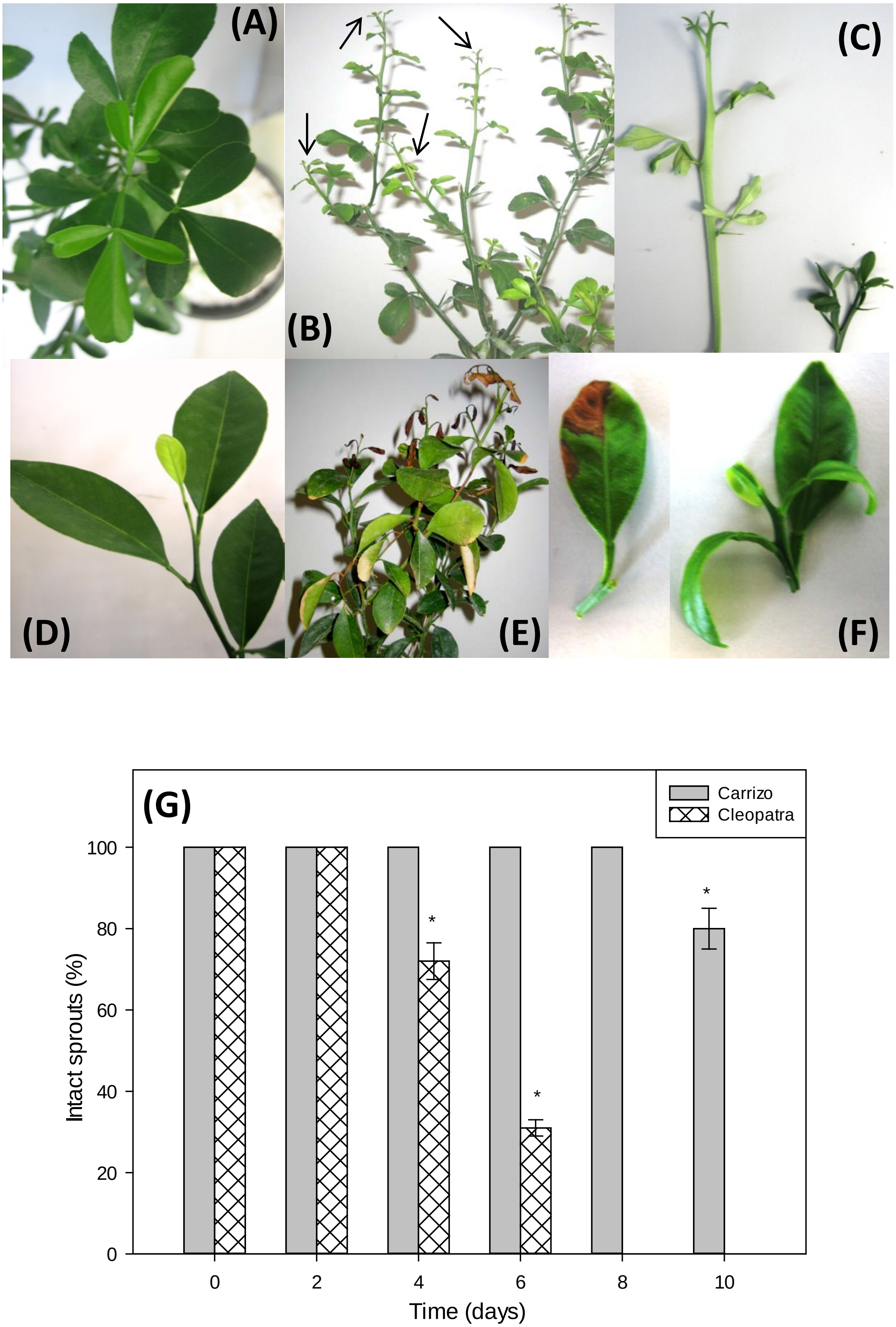

Additional File 3.- (A) Carrizo control sprouts (25ºC); (B) Carrizo plants subjected to heat stress for 10 days; (C) sprouts on control (right) and heat-stressed (left) Carrizo plants; (D) Cleopatra control sprouts; (E) Cleopatra plants subjected to heat stress for 10 days; (F) sprouts on control (right) and heat-stressed (left) Cleopatra plants; (G) Integral sprouts (%) of Carrizo and Cleopatra seedlings subjected to heat stress for 10 days. Asterisks denote statistical significance at $p \leq 0.05$.
